# Hypoxia-inducible factor underlies von Hippel-Lindau disease stigmata

**DOI:** 10.7554/eLife.80774

**Published:** 2022-08-30

**Authors:** Michael Ohh, Cassandra C Taber, Fraser G Ferens, Daniel Tarade

**Affiliations:** 1 Department of Laboratory Medicine and Pathobiology, Faculty of Medicine, University of Toronto Toronto Canada; 2 Department of Biochemistry, Faculty of Medicine, University of Toronto Toronto Canada; University of Pennsylvania United States; Icahn School of Medicine at Mount Sinai United States

**Keywords:** VHL, HIF, PHD, pseudohypoxic diseases, oxygen-sensing, hypoxia

## Abstract

von Hippel-Lindau (VHL) disease is a rare hereditary cancer syndrome that causes a predisposition to renal clear-cell carcinoma, hemangioblastoma, pheochromocytoma, and autosomal-recessive familial polycythemia. pVHL is the substrate conferring subunit of an E3 ubiquitin ligase complex that binds to the three hypoxia-inducible factor alpha subunits (HIF1-3α) for polyubiquitylation under conditions of normoxia, targeting them for immediate degradation by the proteasome. Certain mutations in pVHL have been determined to be causative of VHL disease through the disruption of HIFα degradation. However, it remains a focus of investigation and debate whether the disruption of HIFα degradation alone is sufficient to explain the complex genotype-phenotype relationship of VHL disease or whether the other lesser or yet characterized substrates and functions of pVHL impact the development of the VHL disease stigmata; the elucidation of which would have a significant ramification to the direction of research efforts and future management and care of VHL patients and for those manifesting sporadic counterparts of VHL disease. Here, we examine the current literature including the other emergent pseudohypoxic diseases and propose that the VHL disease-phenotypic spectrum could be explained solely by the varied disruption of HIFα signaling upon the loss or mutation in pVHL.

## Introduction

von Hippel-Lindau (VHL) disease is a hereditary cancer syndrome passed down in an autosomal-dominant fashion. It is named after physicians Eugen von Hippel and Arvid Lindau, who first described families with a genetic predisposition to retinal and central nervous system (CNS) hemangioblastoma, respectively (Lindau, 1904; Davison et al., 1936; Hippel, 1904). VHL disease is rare, afflicting approximately 1 in 35,000 people (Neumann and Wiestler, 1991; Maher et al., 1991). In addition to the retinal and central nervous system hemangioblastoma, other cardinal features of VHL disease include clear-cell renal cell carcinoma (RCC), the commonest form of kidney cancer, and pheochromocytoma, a neuroendocrine tumor derived from adrenal chromaffin cells, in addition to pancreatic, renal and epididymal cysts, endolymphatic sac tumors, and polycythemia (Lindau, 1904 ; Davison et al., 1936; Hippel, 1904).

In 1993, the *VHL* gene was cloned and mutations of the locus were determined to be causative of VHL disease (Latif et al., 1993). By sequencing the *VHL* gene in various families afflicted with VHL disease, a complex genotype-phenotype correlation emerged. First, VHL disease is broadly separated into two types based on the propensity of developing pheochromocytoma where Type 1 patients have a low risk while Type 2 patients have a high risk of pheochromocytoma. Type 1 patients also develop hemangioblastoma and RCC. Type 2 disease is further stratified into Type 2a (high risk of hemangioblastoma with low risk of RCC), Type 2b (high risk of hemangioblastoma and RCC), and Type 2c (only pheochromocytoma without the other disease manifestations). Moreover, *VHL* mutations have been found to be causative of autosomal-recessive familial polycythemia, which is sometimes referred to as Type 3 VHL disease (Table 1). While polycythemia refers to a nonspecific increase in the volume percentage of red blood cells in the blood, also known as hematocrit, this increase in VHL disease and other pseudohypoxic diseases is known to result from excess red blood cell production, termed erythrocytosis. VHL disease Type 1 and 2 are inherited in an autosomal-dominant manner, which predisposes an individual to developing cancer upon the loss of the remaining wild-type allele. In other words, there must be a loss of heterozygosity resulting either from large deletions, mitotic recombination, or hypermethylation of the wild-type allele in a susceptible cell for a tumor to form (Prowse et al., 1997). However, to develop polycythemia associated with VHL disease Type 3, an individual must inherit two mutated *VHL* alleles, meaning it is an autosomal-recessive disease.

**Table 1.** VHL disease classification.

VHL disease					
	Type 1	Type 2A	Type 2B	Type 2C	Type 3
**RCC**	+	-	+	-	-
**Hemangioblastoma**	+	+	+	-	-
**PPGL**	-	+	+	+	-
**Polycythemia**	-	-	-	-	+

**Figure 1.**
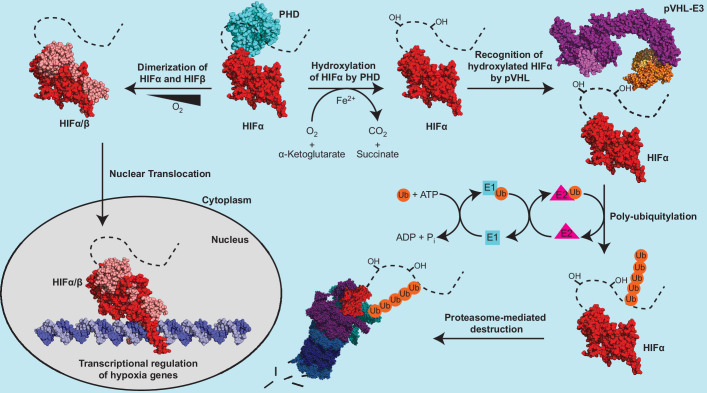
The canonical regulatory components of the metazoan oxygen-sensing pathway. The dotted line connected to the solved HIFα fragment represents the unstructured C-terminal region of HIFα, which contains the oxygen dependent degradation domain. PDB codes of structures used to construct the protein depictions are 4ZP4 (HIF2*α*+HIFβ), 4ZPK (HIF2*α*+HIFβ+Hypoxia-response element), 5L9B (PHD2), 5N4W (Cul2 +Rbx1+EloB + EloC+pVHL), and 3ZRF (EloB +EloC + pVHL). The structural representation of the pVHL-E3 ubiquitin ligase complex was constructed by aligning structures 5N4W and 3ZRF and keeping the most complete model of each duplicated subunit.

The protein product of *VHL* is termed pVHL, which forms an E3 ubiquitin ligase complex (pVHL-E3) with elongin B, elongin C, cullin 2, and Rbx1 (Kamura et al., 1999; Cardote et al., 2017). pVHL serves as the substrate-conferring component of the pVHL-E3 ligase, an integral component in the metazoan oxygen-sensing pathway (Figure 1). The best-characterized substrates of the pVHL-E3 ligase remain the hypoxia-inducible factor (HIF) alpha subunit paralogs HIF1α, HIF2α, and HIF3α. HIFs are heterodimeric transcription factors consisting of an oxygen-labile α subunit and a constitutively expressed β subunit (Figure 1). In the presence of sufficient oxygen, prolyl hydroxylase (PHD) enzymes hydroxylate the conserved proline residues on HIFα, which results in pVHL binding to HIFα and subsequent polyubiquitylation leading to 26 S proteasomal-mediated degradation of HIFα (Figure 1; Epstein et al., 2001; Jaakkola et al., 2001; Ivan et al., 2001; Bruick and McKnight, 2001). Under hypoxic conditions, HIFα remains unhydroxylated, escapes recognition via pVHL, and is therefore not degraded. This pool of stabilized HIFα is able to both dimerize with HIFβ and translocate to the nucleus where it transcriptionally regulates many genes involved in the adaptive hypoxic response including metabolic enzymes, erythropoietin (EPO), and vascular endothelial growth factor (VEGF) (Figure 1; Jiang et al., 1996).

Although the connection between HIF deregulation and VHL disease was readily appreciated, a hypothesis emerged that the complicated genotype-phenotype correlation in VHL disease could not be fully explained by HIF dysregulation alone. This argument implies that pVHL carries out other cellular functions and/or targets other substrates for ubiquitin-mediated degradation that are integral to the manifestation of the full spectrum VHL disease phenotypes. Here, we revisit the available and emerging evidence surrounding the relationship between HIF and VHL disease and propose that HIF dysregulation is the driver of the genotype-phenotype correlation in VHL disease.

## Limited evidence for non-HIF characteristics of VHL disease

The strongest evidence for additional non-HIFα related functions came from early biochemical studies of Type 2c pVHL mutants. Type 2c VHL disease is associated with pheochromocytoma without other disease phenotypes, and several Type 2c pVHL mutants were found to promote HIFα polyubiquitylation at seemingly wild-type levels (Hoffman et al., 2001; Clifford et al., 2001). A second peculiarity centered on Type 1 VHL disease. Despite Type 1 disease driven by mutations predicted to profoundly inactivate pVHL, including nonsense, frameshift and splicing mutations, these patients do not develop pheochromocytoma (Stebbins et al., 1999; Chen et al., 1995; Crossey et al., 1995; Stolle et al., 1998). Thus, pheochromocytoma seemed uncoupled from HIF stabilization (more on this later).

To better understand VHL disease, researchers searched for other targets and/or functions of pVHL. Notably, pVHL has been linked to the regulation of extracellular matrix proteins collagen and fibronectin (Ohh et al., 1998; Kurban et al., 1998a; Grosfeld et al., 2007; Stickle et al., 2004; Kurban et al., 1998b), microtubules (Thoma et al., 2009; Hergovich et al., 2006; Hergovich et al., 2003), primary cilia (Montani et al., 2010; Esteban et al., 2006), NFκB (Cummins et al., 2006; Yang et al., 2007), Human Antigen R (HuR) (Danilin et al., 2009; Massfelder et al., 2004), AKT kinase (Guo et al., 2016), p14/ARF (Minervini et al., 2015; Lai et al., 2011), and ZHX2 (Zhang et al., 2018).

A subset of these potential pVHL regulatory targets, including AKT, are proposed to interact with pVHL in a hydroxylproline-dependent manner following PHD-mediated hydroxylation. Notably, a systemic attempt to detect PHD-mediated hydroxylation of 24 non-HIFα substrates failed to reproduce and confirm previously published findings (Cockman et al., 2019). Further, a list of potential pVHL substrates, including fibronectin, collagen, tubulin, HuR, and p14/ARF, have been identified as pVHL interactors in high throughput studies with additional low-throughput supporting evidence in the BioGRID database (Oughtred et al., 2021). Each of these potential interactors, however, have only been identified in a single high-throughput experiment and all but HuR have been identified by the same high-throughput study (Lai et al., 2011; Oughtred et al., 2021). In contrast, the HIFα substrates of pVHL have been identified by numerous high-throughput and low throughput studies (143 identify HIF1α and 19 identify HIF2α in the BioGRID database) demonstrating a disparity in the confidence of the interactions (Oughtred et al., 2021). Thus, strong evidence of non-HIFα related *bona fide* biological functions of pVHL remains lacking.

### Evidence for a HIF-centric model of VHL disease

#### Clinical evidence

**Figure 2.**
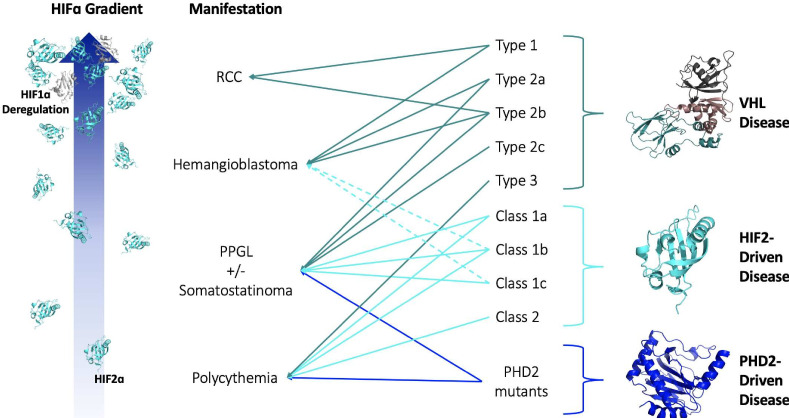
HIF-centric unifying model of VHL and other pseudohypoxic diseases. See text for details. VHL, Elongin B, Elongin C (PDB:1VCB); HIF2α C-Terminal PAS Domain (PDB:3F1P); HIF1α C-Terminal PAS Domain (PDB:4ZPR); PHD2 Catalytic Core (PDB: 5L9B).

Since the association of pVHL mutations with VHL disease, clinicians noted that largely unique groups of pVHL mutations were associated with the different types of VHL disease. It has been generally accepted that mutations that more markedly impact the ability of pVHL to degrade HIFα result in more ‘severe’ VHL disease phenotypes. Patients with large deletion and frameshift mutations generally develop Type 1 VHL disease, while patients with missense mutations develop Type 2 disease (Chen et al., 1995; Stolle et al., 1998; Maher et al., 1996). This concept can be further extrapolated to explain the heterogeneity of phenotypes within Type 2 VHL disease. Type 2c pVHL mutants, for example, cause exclusively pheochromocytoma and have been shown to degrade HIFα nearly as well as wild-type pVHL (Hoffman et al., 2001). Further, some pVHL mutations are associated with incomplete phenotypic penetrance, which has even been observed among members of the same VHL disease family. Thus, we propose that the various clinical phenotypes associated with VHL disease require different ‘doses’ of HIF. From polycythemia to pheochromocytoma to hemangioma/hemangioblastoma to RCC, a greater dose of HIF is required for disease onset. Inter-family heterogeneity among VHL disease patients reflects a differential loss of pVHL function — a mutation that completely abrogates the ability of pVHL to negatively regulate HIF leads to a greater HIF dose and more severe phenotype than mutations that only mildly impair pVHL function. Lastly, observed intra-family heterogeneity supports the idea that different phenotypes associated with VHL disease exist on a gradient of increasing HIF dose. Some individuals in a family, due to environmental, metabolic, and/or genetic factors, experience greater stabilization of HIF than other relatives with the same mutation, which leads to the development of phenotypes otherwise associated with greater loss of pVHL function (Figure 2).

pVHL Y98 and Y112 are recognized as residues of interest as different missense mutations at these sites can elicit drastically different phenotypes. Both the Y98H and Y112H VHL mutations are classically associated with VHL Type 2a disease with a high penetrance of pheochromocytoma, but while the Y112H mutation appears to result in hemangioma/hemangioblastoma in only 20% of cases, the Y98H mutation displays a greater penetrance at ~50% (Brauch et al., 1995; Chen et al., 1996). This observation is also consistent with the fact that patients with the Y98H pVHL mutation are diagnosed earlier (mean of 19.7 years-old) than patients with the Y112H pVHL mutation (28.8 years-old) (Nielsen et al., 2011a). The fact that hemangioma/ hemangioblastoma penetrance lags behind pheochromocytoma penetrance in VHL disease patients with the Y112H mutation points to the idea of a gradient, where only a subset of these Type 2a patients develop the cardinal feature of vascular hemangioma/hemangioblastoma tumors.

In addition, a small number of patients with Type 2a pVHL mutants also develop RCC. Indeed, Type 2a VHL disease is classically defined as a ‘low’ chance of RCC rather than ‘no’ chance. A study of 16 families harboring the Y98H mutation found that some affected individuals exhibited symptoms consistent with Type 1 or 2b VHL disease. Out of 116 affected family members, 73 developed pheochromocytoma while 4 developed RCC (Brauch et al., 1995). Two individuals developed RCC in addition to pheochromocytoma (Type 2b) and two solely developed RCC (Type 1). Similarly, ~3% of individuals with the Y112H mutations also develop RCC (Nielsen et al., 2011b). In comparison, in a family harboring Y112N, 7 individuals developed RCC and only one developed pheochromocytoma, suggesting Y112N presents more commonly as Type 1 VHL disease (Bradley et al., 1999).

While Type 2c VHL disease is associated with pheochromocytoma, Type 2c mutations can sometimes ‘spillover’ and also cause hemangioma/hemangioblastoma. One example is the classic Type 2c VHL mutant V84L, which is associated solely with pheochromocytoma in most patients but in some cases, vascular tumors, including the possibility of spinal hemangioblastoma, and renal cysts are observed (Abbott et al., 2006). Similarly, the Type 2c VHL mutant G93S was linked with pheochromocytoma across multiple generations in a family with VHL disease, but further diagnostic workup revealed two retinal angiomas in the index patient and bilateral renal cysts in the index patient’s father (Schreinemakers et al., 2007). While renal cysts can be found in a population not carrying pVHL mutations, it is important to note that this could be evidence of progression of Type 2c VHL disease as individuals age.

As mentioned above, Type 2c VHL disease has perplexed the field as Type 2c pVHL mutants appear to regulate HIFα in a manner similar to wild-type pVHL, which would suggest that pheochromocytoma is uncoupled from HIFα degradation (Hoffman et al., 2001; Clifford et al., 2001). For example, a patient with bilateral pheochromocytoma and no other VHL-related symptoms was found to harbor a Y175C VHL mutation. When compared to wild-type pVHL, Y175C pVHL was able to degrade HIF efficiently under normoxic conditions (Astapova et al., 2018). But while in vitro studies failed to associate pheochromocytoma pathogenesis with HIF stabilization in the context of VHL disease, clinically observed *EPAS1* (encodes HIF2α) and *EGLN1* (encodes PHD2) mutations that result in HIF2α stabilization are now known to cause disease phenotypes that partially overlap with the VHL disease spectrum. Of particular interest, pheochromocytoma carcinogenesis is clinically associated with PHD2 loss-of-function mutations and HIF2α gain-of-function mutations in addition to pVHL loss-of-function mutations (Gardie et al., 2014; Ladroue et al., 2008; Därr et al., 2016; Taieb et al., 2016; Tarade et al., 2018). Therefore, it is possible that previous experiments suggesting the near wild-type activity of Type 2 c pVHL mutants lacked the sensitivity to assess differences in HIFα stabilization relative to wild-type pVHL that are biologically relevant. It is also possible that there is a more complex interplay between the relative stabilization of the different HIFα paralogs, which may be differentially affected by certain pVHL mutations.

HIF2-driven disease is particularly interesting because like VHL disease, it also presents as a gradient with escalating phenotypes comparable to Type 3, Type 2c, and even Type 2a VHL disease. Patients with mutations in *EPAS1*, the gene encoding HIF2α, have been reported to develop polycythemia and neuroendocrine tumors in dozens of patients (Därr et al., 2016; Taieb et al., 2016; Tarade et al., 2018). HIF2-driven disease can be segregated into two broad classes: Class 1 characterized by the presence of pheochromocytoma and/or paraganglioma (PPGL) with or without polycythemia, and Class 2 in which patients present solely with polycythemia (Tarade et al., 2018). Class 1 is further divided into three sub-classes where Class 1a presents with PPGL, somatostatinoma, and polycythemia, Class 1b presents with PPGL and polycythemia, and Class 1c presents with PPGL exclusively (Table 2). More rarely, patients with gain-of-function HIF2α mutations also present with hemangiomas and hemangioblastoma. While there is only a single case of hemangioblastoma linked to a HIF2α gain-of-function mutation, a unique and uncharacterized double F374Y and Q561K missense mutation, (Taieb et al., 2016) the clinical literature includes three reports of patients with HIF2-driven disease developing hemangiomas of the retina, liver, and cranial vault (Pacak et al., 2013; Dmitriev et al., 2020; Buffet et al., 2014). All three of these patients also developed polycythemia and pheochromocytoma/paraganglioma and were determined to have a HIF2α gain-of-function mutation otherwise associated with a complete penetrance of PPGL. While there is less clinical evidence of HIF2α driving hemangioma, relative to PPGL, liver hemangiomas associated with loss-of-pVHL mouse models can be rescued by co-deletion of HIF2α or HIFβ, which provides further evidence for the gatekeeping role of HIF2α in hemangioma and hemangioblastoma (Rankin et al., 2008; Rankin et al., 2005). Much like Type 2c VHL disease, Class 1a/b HIF2-driven disease can sometimes ‘spillover’ and trigger the formation of vascular hemangioma tumors. The remarkable fact that HIF2α gain-of-function mutations can phenocopy Type 2a VHL disease provides strong evidence for the role of HIF2α in Type 2c VHL disease. Not only do some canonical Type 2c VHL mutations also cause hemangiomas, a phenotype strongly linked with HIF2α signaling in Type 2a VHL disease, some VHL disease patients concurrently develop polycythemia, pheochromocytoma, and somatostatinoma, which matches the phenotypic profile of Class 1a HIF2-driven disease (Karasawa et al., 2001).

**Table 2.** HIF2-driven disease classification.

HIF2-driven disease				
	Class 1A	Class 1B	Class 1C	Class 2
**PPGL**	+	+	+	-
**Somatostatinoma**	+	-	-	-
**Polycythemia**	+	+	-	+

PHD2 mutations have been widely found to cause polycythemia. More rarely, PHD2 mutations, like PHD2 H374R and PHD2 A228S, trigger development of pheochromocytoma and/or paraganglioma (PPGL) in addition to polycythemia (Gardie et al., 2014; Ladroue et al., 2008; Yang et al., 2015). The patient harboring the H374R mutant originally presented with erythrocytosis, but a tumor matching the characteristics of a paraganglioma was later diagnosis at 43 years old (Ladroue et al., 2008) This individual displayed loss of the remaining wild-type *EGLN1* allele. This observed loss of heterozygosity suggests that PHD2 plays a tumor suppressor role in chromaffin cells and the closely related paraganglia cells that are transformed in paraganglioma. Other reports of PHD2 mutation in polycythemia note that patients inherit a single mutated allele (Percy et al., 2006; Percy et al., 2007; Barradas et al., 2018). This could imply that haploinsufficiency of wild-type PHD2 might be enough to induce symptoms. Indeed, deletion of a single PHD2 allele or knock-in of a single mutant PHD2 allele in mice successfully recapitulates polycythemia, which was also found to be dependent on HIF2α (Arsenault et al., 2013). These cases also suggest mutations of *EGLN1* may have a limited phenotypic range compared to VHL and HIF2-driven disease. The loss of PHD2 function may be compensated for by the other PHD paralogs, PHD1 and PHD3, preventing most instances of tumor formation but not all. Indeed, there has been one documented case of a disease-causing PHD1 mutation. This mutant, S61R, induced erythrocytosis development by age 6 and recurrent PPGL at age 14 (Yang et al., 2015).

There have also been documented cases of pVHL mutant-induced polycythemia (Type 3 VHL disease). R200W and H191D have both been identified as autosomal-recessive pVHL mutations that cause polycythemia (Pastore et al., 2003; Ang et al., 2002). And similarly to the role of HIF2α in liver hemangioma pathogenesis in loss-of-pVHL mouse models, inhibition of HIF2α rescues polycythemia in mouse models of polycythemia driven by the R200W mutation (Ghosh et al., 2021; Hickey et al., 2007; Ghosh et al., 2018).

While pVHL induced polycythemia is generally inherited in an autosomal-recessive manner, a study investigating eight children with polycythemia revealed one case in which a family harbored a heterozygous VHL mutant inducing a phenotype. Two siblings in the family were found to be heterozygous for D126Y and had increased levels of hematocrit and erythropoietin. One of the siblings had a history of tumors, developing a pulmonary angioma at age 10 and a renal subcapsular hemangioma at age 15. This would indicate that pVHL D126Y straddles the line between Type 3 and Type 2a disease. All the patients in this study were young (10–19 years old; Pastore et al., 2003); therefore, it remains unclear whether these patients will develop tumors later in life.

Just like VHL disease, HIF2α and PHD2 mutations can present a wide range of phenotypes where mutations that most stabilize HIF2α cause the worst phenotypes (more on this later). Thus, mutations in these three separate components of the HIFα-degradation pathway — substrate (HIF2α), hydroxylase (PHD2), and E3 ubiquitin ligase (pVHL) — all present with similar phenotypes. The common factor in the disruption of these three components of the pathway is the ultimate stabilization of HIFα, in particular HIF2α, and the correlation between the severity of disease and the degree of disruption to the HIFα degradation pathway naturally suggest that the causative factor is HIFα (Figure 2).

### Biochemical evidence

The clinical evidence that pseudohypoxic diseases resulting from mutations in multiple components of the metazoan oxygen-sensing pathway have similar phenotypes to VHL disease suggests a common mechanism underlying these diseases. The notion that the magnitude of HIF stabilization is the sole mechanism that gives rise to the various phenotypic spectrum of VHL disease would infer that the downstream pathways and gene regulation by HIF have sufficient complexity to explain the multifaceted genotype-phenotype relationship. The oxygen-sensing pathway in humans is canonically comprised of three PHD paralogs (PHD1, PHD2, PHD3), three HIFα paralogs (HIF1α, HIF2α, HIF3α), two HIFβ paralogs (ARNT and ARNT2), and a single pVHL-E3 ligase (Figure 1; Kaelin and Ratcliffe, 2008). The complexity of the pathway is also influenced by the differential behavior of the paralogs of each component; each PHD paralog has varying hydroxylation activity toward the HIFα paralogs (Appelhoff et al., 2004), each HIFα paralog has 2 hydroxylation sites with varying oxygen-dependent hydroxylation sensitivities (Tarhonskaya et al., 2015), HIF3α has multiple splice variants (Maynard et al., 2003) and the HIFα paralogs regulate partially overlapping as well as unique sets of genes (Schödel et al., 2011). Thus, the complexity of the oxygen-sensing pathway allows for mutations to have differential effects on HIF stabilization and the expression of downstream genes and pathways depending on the specific component of the pathway affected and the severity of the mutation.

The disease-associated pVHL mutations can be segregated into three categories; those that affect pVHL protein-protein interfaces, those that affect pVHL stability and folding, and those that completely inactivate pVHL through gross truncation or deletion. All VHL disease-associated mutations are observed to stabilize the HIFα substrates through reductions in polyubiquitination of the three HIFα paralogs. However, the degree to which the HIFα substrates are stabilized varies between the different types of mutations (Rechsteiner et al., 2011), VHL Type 1 mutations in which pVHL is grossly or completely inactivated through truncation or frameshift result in maximal stabilization of HIFα whereas VHL Type 2 mutations affecting the folding, stability, or substrate-binding interface of pVHL have an intermediate effect on HIFα stabilization due to the remaining pVHL activity (Rechsteiner et al., 2011; Knauth et al., 2006; Hacker et al., 2008; Forman et al., 2009). Among the various sub-types of VHL disease, multiple biochemical studies demonstrate that Type 2b mutations result in greater HIF stabilization than Type 2a mutations (Knauth et al., 2009; Li et al., 2007). While multiple studies suggest that VHL Type 2c mutations do not negatively impact the ability to regulate HIFα, other studies argue that Type 2c mutations result in an unstable pVHL-E3 ubiquitin ligase complex and a mild stabilization of HIF1α (Knauth et al., 2009). These data indicate that more deleterious mutations in pVHL lead to a correspondingly more HIFα stabilization, which appears to correlate with disease severity.

Numerous missense mutations in the C-terminal prolyl-hydroxylation site in the oxygen-dependent degradation domain of HIF2α have been reported to cause polycythemia, pheochromocytoma, paraganglioma, somatostatinoma, hemangioma, and in one case hemangioblastoma (Taieb et al., 2016; Tarade et al., 2018). These phenotypes linked to HIF2α mutations have been collectively referred to as HIF2-driven disease, which has been broadly separated into two classes where Class 1 is causative of neuroendocrine tumors including paraganglioma, pheochromocytoma, and somatostatinoma (as well as possible hemangioma/hemangioblastoma) with or without polycythemia while Class 2 is causative of only polycythemia (Tarade et al., 2018). Notably, the HIF2-driven disease classes are linked to unique sets of HIF2α mutations defining a genotype-phenotype correlation similar to VHL disease (Tarade et al., 2018). These HIF2α mutations reduce the affinity of hydroxylated HIF2α to pVHL with increasing reductions in binding affinity correlated to increasing severity of the disease phenotype with Class 1 mutations being more deleterious than Class 2 mutations (Tarade et al., 2018).

As mentioned above, there are also reports of mutations in PHD2 and one mutation in PHD1, which cause deficiencies in the hydroxylation of HIFα paralogs and therefore regulation of HIFα. These PHD mutations are causative of polycythemia and PPGL (Ladroue et al., 2008; Yang et al., 2015; Ladroue et al., 2012). Studies have shown that PHD2 mutants have an impaired ability to reduce HIF1α and HIF2α levels when transfected and expressed in mammalian cells (Yang et al., 2015; Percy et al., 2006; Ladroue et al., 2012). Further, the PPGL-associated H374R PHD2 mutation was found to severely abrogate the capacity for HIF2α regulation relative to most mutations that cause only polycythemia (Ladroue et al., 2012). Thus, there are similarities between VHL disease, HIF2-driven disease and the pseudohypoxic disease caused by PHD mutations, which are driven by a common mechanism, the dysregulation of HIFα paralogs, particularly HIF2α. The inhibition of hydroxylation or abrogation of recognition of hydroxylated HIF2α by pVHL leading to the dysregulation of HIF2α may be sufficient to explain the disease phenotypes of all three pseudohypoxic diseases.

However, mutations in HIF2ɑ and PHD2 have not been observed to be causative of RCC indicating that HIF2α stabilization independent of improper HIF1α regulation is not sufficient to drive the development of RCC. This is interesting as in vivo experiments using nude mouse xenograft assays provide some insight into the role of HIF2α in RCC. Tumors extracted from mice injected with 786-O cells co-expressing Type 2a pVHL mutant (Y98H or Y112H) and a stabilized form of HIF2α (proline hydroxylation site double mutant, P405A and P531A) were observed to have increased RCC tumor mass as compared to mice injected with cells lacking the stabilized HIF2α. The opposite effect was observed in nude mouse xenograft assays where mice were injected with 786-O cells co-expressing Type 2b mutant pVHL (Y98N or Y112N) and a HIF2α-targeted shRNA to knockdown HIF2α expression. Additionally, a ~50% decrease in the number of mice with tumors relative to mice injected with cells expressing the control shRNA was observed with the Y112N pVHL mutant. These observations suggested that increases to intracellular HIF2α concentration promotes RCC tumor growth and decreases to the intracellular HIF2α concentration inhibits tumor growth and initiation (Li et al., 2007). A recent mouse model system using induced gene deletion of combinations of VHL, HIF1α, HIF2α, Trp53, and Rb1 revealed that RCC tumor formation is strongly dependent on HIF1α (Hoefflin et al., 2020). VHL^Δ/Δ^HIF1α^Δ/Δ^ mice (Note: all mice in the study were also Trp53 ^Δ/Δ^Rb1^Δ/Δ^) showed a drastic reduction in tumor frequency relative to VHL^Δ/Δ^ mice. VHL^Δ/Δ^HIF2α^Δ/Δ^ mice by comparison only had a moderate reduction in tumor frequency relative to VHL^Δ/Δ^ mice (Hoefflin et al., 2020). It was also observed that the stabilization of both HIF1α and HIF2α in VHL^Δ/Δ^ mice promotes the clear cell phenotype of tumor cells while tumor cells from VHL^Δ/Δ^HIF1α^Δ/Δ^ mice and VHL^Δ/Δ^HIF2α^Δ/Δ^ mice had reduced frequency of the clear cell phenotype (Hoefflin et al., 2020). These data suggest a more complex relationship between the relative expression of HIF1α and HIF2α in RCC. Taken together, the deregulation of HIF2α expression alone is sufficient to explain a subset of VHL disease phenotypes (i.e. Type 2A, 2C, and 3 VHL disease), and there are data to suggest that the remaining phenotypes related to RCC (i.e. Type 1 and 2B VHL disease) could be explained by the co-involvement of HIF1α and HIF2α.

### Dual role of HIF1α and HIF2α in kidney cancer

Given the clear clinical and biochemical evidence pointing towards HIF2α driving polycythemia and PPGL, the balance of HIF-dependent or HIF-independent pVHL roles in RCC instead becomes the focus. Unlike the other cardinal stigmata associated with VHL disease, loss-of-function PHD2 mutations or gain-of-function HIF2α mutations have not been observed in RCC (Morris et al., 2009; Astuti et al., 2011). This suggests that either the dysregulation of HIF-independent VHL functions plays a role in RCC or the coupled deregulation of HIF1α and HIF2α is uniquely required for RCC.

Some researchers argue that HIF2α is the main driver of RCC arising from VHL disease. Human RCC 786-O cell line lacks both pVHL and HIF1α while overproducing HIF2α, making them useful for studying VHL disease. 786-O cells transfected with HIF2α P405A/P531A double mutants (unable to be hydroxylated by PHDs) can induce tumors in nude mice when implanted. Growth of these tumors can be reduced via siRNA-mediated knockdown of HIF2α, but this defect cannot be rescued by expression of a basic helix-loop-helix (bHLH) HIF2α mutant incapable of DNA binding (Kondo et al., 2003; Kondo et al., 2002; Zimmer et al., 2004). This suggests HIF2α suppression is necessary for pVHL tumor suppression and that HIF2α is, therefore, a driver of RCC. There is also evidence that the loss of chromosome 14q, harboring the HIF1α gene, is a hallmark of RCC metastasis and results in poor prognosis for patients (Shen et al., 2011; Monzon et al., 2011; Turajlic et al., 2018). In addition, downregulation of HIF1α in kidney cancer cells promotes tumor growth, while supplementing HIF1α deficient cell lines with HIF1α leads to tumor regression (Shen et al., 2011). These data argue that HIF1α may have a tumor suppressive role as low levels or loss of HIF1α is associated with tumor progression while HIF1α supplementation reduces tumor growth. Altogether, a more complicated relationship between HIF1α and HIF2α in their roles in RCC development is suggested.

Other convincing data on the RCC tumorigenesis process comes from recent genetic mouse models. Through the inactivation of pVHL via conditional knockout in collecting ducts and distal tubules, mice develop overt carcinoma (Pritchett et al., 2015). Deletion of HIF1α but not HIF2α completely abolishes tumorigenesis in these models (Pritchett et al., 2015). Further, the forced expression of HIF1α leads to the formation of dysplasia, cysts, and precancerous lesions while HIF2α expression results in only minor cellular phenotypes (Fu et al., 2011; Fu et al., 2015; Fu et al., 2013). Most recent models highlight the role of both HIF1α and HIF2α in RCC (Hoefflin et al., 2020; Pritchett et al., 2015; Fu et al., 2015; Schönenberger et al., 2016). Further, common mutations in other tumor suppressors, such as SWI/SNF protein PBRM1, have been linked to the amplification of HIF1α signaling (Nargund et al., 2017; Gao et al., 2017).

In conjunction, genetic mouse models and xenograft models suggest a unique role for HIF1α in the development of RCC. HIF1α is absolutely required for the initial dysplasia and cyst formation that defines the early stages of tumorigenesis while both HIF1α and HIF2α are required for overt carcinoma. In an established tumor, like those used to create the cell lines used in xenograft studies, HIF1α may suppress tumor growth and/or metastasis, and the loss of HIF1α therefore may induce worse prognoses. At minimum, HIF1α is expendable once a tumor is established. This model is further supported by the staining of human tissues, where HIF1α and carbonic anhydrase IX (CAIX) staining is prominent in precancerous lesions and cysts while HIF2α is expressed most in overt carcinoma (Mandriota et al., 2002). It is possible therefore, HIF1α first acts as an oncogene and tumor initiator but following a switch in HIF expression, it acts as a tumor suppressor, preventing disease progression.

Importantly, this model of RCC does not need to appeal to HIF-independent functions of pVHL to explain the broad process of tumorigenesis. For example, multiple studies have linked dysregulation of fibronectin deposition to the loss of pVHL function (Ohh et al., 1998; Tang et al., 2006). The loss of pVHL is associated clinically with both the stabilization of HIF1 and the inappropriate cytoplasmic accumulation of fibronectin in the earliest lesions and cysts that appear during the RCC transformation process (Mandriota et al., 2002). So while loss of pVHL function is correlated with the dysregulation of some matrix proteins like fibronectin and collagen, only HIF stabilization is causally linked with the development of VHL disease. Other functions of pVHL may indeed play a role and impact cellular phenotypes, but the transformation process itself can be driven by the coupled, perhaps interlinked, regulation of HIF1α and HIF2α. This should be noted and taken into consideration as novel HIF-PHI (Prolyl-Hydroxylase Inhibitor) drugs begin to gain widespread approval and use.

## Other considerations

### Distinguishing SNPs from disease-causing mutations

When studying mutations in *VHL*, *EGLN1*, and *EPAS1*, it is imperative to exclude single nucleotide polymorphisms (SNP), hypomorphic alleles, and passenger mutations observed in individuals presenting with pseudohypoxic diseases when other verified disease-causing mutations are also present. These apparent mutations are not causative of disease and thus can confound genotype-phenotype relationships. One example of this is the P25L VHL mutant. This mutation was found in conjunction with another VHL mutant P86R in a patient diagnosed with VHL disease. P86R has been identified as a disease-causing mutation, suggesting P25L is a rare polymorphic allele of *VHL* rather than a disease-causing mutation (Rothberg et al., 2001). When considering that P25L localizes to the N-terminal acidic domain, which has no known role in regulating HIF, we argue that this mutation should be excluded from genotype-phenotype studies.

Although most reported HIF2α mutations cluster around the C-terminal hydroxylation site, P531, a handful impact residues with no clear role in HIFα regulation. Although it is tempting to speculate about new modes of HIF regulation, most of these mutations present as rare SNPs, including F374Y (rs150797491), M538I (rs61757375), and T519M (rs377001303). Indeed, by comparing the frequency of the previously mentioned F374 allele in cohorts with and without PPGL, a lifetime penetrance of only 5% was predicted (Dwight et al., 2021). This is consistent with the observation that in familial cases, the F374Y mutation is uncoupled from the PPGL phenotype (Lorenzo et al., 2013). Thus, mutations with well-characterized biochemical or molecular mechanism and firmly associated with a particular phenotype ought to be the focus of future genotype-phenotype studies and care should be taken to assess whether novel mutations are truly disease causing or not.

### The intersection of genetic lesions and environment

In addition to the presence of SNPs mis-identified as bona fide disease-causing mutations, those investigating the genetic basis of pseudohypoxic diseases must also keep in mind the interaction between genetic lesions and the environment. We argue that environmental factors can complicate disease presentation. Generally, hypoxemia associated with living at altitude or a chronic health condition like cyanotic heart disease is a known risk factor for developing a carotid body paraganglioma (Lack, 1977; Lack, 1978; Arias-Stella and Valcarcel, 1976; Heath et al., 1970). Further, it has been reported that patients with germline mutations in *SDHD*, the gene encoding succinate dehydrogenase subunit D, are more likely to present with multiple PPGL tumors if they live at altitude than patients living at sea level (Astrom et al., 2003). The implication is that low levels of hypoxemia or systemic hypoxia increases HIF2α activity, which drives hyperplasia and increases the odds of multiple transformation events occurring over time (Astrom et al., 2003). It is also broadly accepted that as an individual ages, the risk of cancer increases due to various factors such as telomere shortening and accumulated exposure to carcinogens (Anisimov, 2003). Patients with pseudohypoxic disease therefore may develop additional symptoms as they age, shifting their disease presentation into a different class or type.

These observations may help deconvolute the spectrum of phenotypes relating to HIF2 disease-causing mutations. Although there is a strong genotype-phenotype relationship regarding cancer-causing and non-cancer-causing HIF2α mutations, patients presenting with PPGL display a wide range of clinical outcomes, ranging from a single PPGL tumor and no polycythemia to multiple PPGL tumors with polycythemia to PPGL tumors comorbid with both polycythemia and somatostatinoma (Tarade et al., 2018). As there is no clear genotype-phenotype relationship governing these possible clinical outcomes, we instead propose that environmental factors governing overall oxygen saturation likely play a key role (Tarade et al., 2018).

Indeed, even the boundary between cancer-causing and non-cancer-causing mutations in HIF2-driven disease may become blurred as more patient data becomes available. Recently, the HIF2α D539N mutation has been reported in patients with both PPGL and polycythemia (i.e. Class 1 phenotype) and patients with only polycythemia (i.e. Class 2 phenotype), suggesting that this mutation can cause either Class 1 and 2 HIF2-driven disease in different individuals (Oliveira et al., 2018; Pacak et al., 2014; Pang et al., 2019). Although it is possible that the patient diagnosed with polycythemia may go on to develop PPGL, as PPGL diagnosis always lags polycythemia diagnosis in these patients, it is also possible that this mutation straddles the line between polycythemia and PPGL and environmental factors may play a deciding role in the degree of penetrance. Combined, this supports our hypothesis that the presentation of VHL disease and other pseudohypoxic disorders depends on the degree of HIF2α activation, HIF1α dysregulation and the influence of environment and other defects in cardiovascular function that can impart a degree of hypoxemia.

## Next steps

Fully elucidating molecular mechanism underlying VHL disease remains difficult given a dearth of relevant culture and animal models. *VHL*-null kidney cancer cell lines led to the discovery of the role of pVHL and prolyl hydroxylases in negatively regulating HIF, but overexpression of mutant pVHL, PHD2, or HIF2α remains a blunt tool that is not well-suited for differentiating different disease classes. Without a cell culture system relevant to the RCC, hemangioma or PPGL carcinogenesis process, the principal readout available is overall stabilization of HIF2α, a decidedly qualitative result. Thus, the study of pseudohypoxic disease, which benefits greatly from rich clinical data, requires a model for the one-to-one comparison of pVHL, HIF2α, and PHD2 mutants. If our HIF-centric model of pseudohypoxic disease holds then we would expect that Type 2c pVHL mutants would result in similar HIF2α stabilization as Class 1 HIF2α mutants or cancer-causing PHD2 mutants; Type 2a pVHL mutants would result in greater stabilization of HIF2α than either Type 2c pVHL mutants or Class HIF2α mutants; and Type 2b pVHL mutants would result in even greater stabilization of HIF2α.

Any model developed to assess the HIF-centric model of VHL disease, where increasing dysregulation of HIF corresponds to a wider range of phenotypes, will need to be able to assess overall HIF stabilization in a system that includes all components of the PHD-pVHL-HIF axis. Further, these components will need to reflect physiologically relevant concentrations. Lastly, quantitative readouts directly related to the stabilization of HIF2α and/or HIF1α would be required.

Although possible to recapitulate the PHD-pVHL-HIF axis in living systems, the ability to introduce disease-causing mutations without over-expressing one component is limited. CRISPR-mediated gene editing of endogenous *VHL*, *EGLN1*, or *EPAS1* loci remains one possibility to directly compare the impact of disease-causing mutations on the regulation of cellular HIF pools. By comparing this cellular data with known clinical phenotypes, the HIF-centric model could be directly addressed.

Further, there is some limited success in recapitulating pseudohypoxic disease phenotypes in mice (Rankin et al., 2005; Hickey et al., 2007; Wang et al., 2019; Tan et al., 2013). Generation of transgenic mice with representative *VHL*, *EGLN1*, and *EPAS1* mutants thus represents another avenue by which the HIF-centric model can be tested. So far, polycythemia has been recapitulated in transgenic mice with either *VHL* loss or *EPAS1* gain-of-function mutation. Importantly, a gradient in EPO levels could be discerned between heterozygous and homozygous HIF2α gain-of-function mutation, suggesting this is a sensitive and quantitative readout (Tan et al., 2013). Unfortunately, it’s been difficult to generate a mouse model of pseudohypoxic pheochromocytoma or paraganglioma (Lussey-Lepoutre et al., 2018). One exception is the work of the Bishop group where the inactivation of PHD2 in the Type 1 cells of carotid body resulted in the formation of paraganglioma (Fielding et al., 2018).

Another possible model for testing the HIF-centric hypothesis is the in vitro reconstitution of the entire pVHL-PHD-HIF axis. Purification of PHD2, pVHL-E3 ubiquitin ligase complex, and fluorescein-labeled HIF2α would allow for the real-time monitoring of HIF2α ubiquitylation and degradation. By substituting wild-type components with disease-associated mutations, the level of HIF2α stabilization could be precisely assessed and compared to known clinical phenotypes.

Lastly, the development and clinical evaluation of a specific HIF2α inhibitor, Belzutifan, represents another opportunity to assess the necessity of HIF2α activity for the various clinical manifestations associated with VHL disease (Cho et al., 2016; Chen et al., 2016). Recently, Belzutifan received approval in the US for treatment of RCC, hemangioblastoma, and pancreatic neuroendrocrine tumors (pNET) associated with VHL disease following a successful phase 3 clinical trial (Fallah et al., 2022). A small number of pheochromocytoma tumors in that clinical trial meant that the critical requirement of HIF2α signaling for VHL disease-associated pheochromocytoma could not be assessed, but a single-patient clinical trial of a patient with Pacak-Zhuang syndrome (another name for Class 1a/b HIF2-driven disease) revealed that Belzutifan treatment can result in the shrinkage of paraganglioma tumors and the resolution of polycythemia (Kamihara et al., 2021). Expanded studies evaluating the efficacy of Belzutifan in PPGL tumors associated with VHL disease and HIF2-driven disease will allow for the exploration of the necessity of HIF2α for PPGL in different genetic contexts.

### Conclusions

VHL disease is a rare hereditary cancer syndrome that causes a perplexingly wide variety of phenotypes. Despite a myriad of suggested HIF-independent functions for pVHL, we propose that the complexities of VHL disease can be explained solely within the context of HIF dysregulation. The most striking supportive evidence of this notion is the genetics of HIF2- and PHD2-driven diseases in which mutations in these critical components of the metazoan oxygen-sensing pathway have been found to cause similar phenotypes to those observed in VHL disease. Disease caused by mutations in HIF2α, PHD2, and pVHL all stabilize HIF to cause an inappropriate hypoxic response. A gradient can be observed in which mutations that most stabilize HIF induce the most severe phenotypes (Figure 2). A complex molecular interplay between HIF1α and HIF2α is likely required for the development of RCC, which in turn can explain some puzzling aspects of VHL disease. Further investigations surrounding the complexities of VHL disease should not overlook, but rather return to, the one *bona fide* function of pVHL to interrogate how differing *VHL* mutations affect the degree of stabilization of HIFα.
